# Toward Complementary Food Hygiene Practices among Child Caregivers in Rural Malawi

**DOI:** 10.4269/ajtmh.18-0639

**Published:** 2019-06-24

**Authors:** Kondwani Chidziwisano, Jurgita Slekiene, Save Kumwenda, Hans-Joachim Mosler, Tracy Morse

**Affiliations:** 1 Department of Environmental Health and WASHTED Centre, Polytechnic, University of Malawi, Blantyre, Malawi;; 2 Department of Civil and Environmental Engineering, University of Strathclyde, Glasgow, Scotland;; 3 Eawag, Swiss Federal Institute of Aquatic Science and Technology, Dübendorf, Switzerland

## Abstract

Despite being preventable, foodborne diseases remain a global health challenge. Poor food hygiene practices such as improper handling of kitchen utensils are among the major causes of diarrhea transmission. A formative study was conducted in Malawi to inform an intervention design to promote complementary food hygiene practices. An assessment of contextual and psychosocial factors for behavior change was conducted using Risk, Attitude, Norms, Ability, and Self-regulation model. We conducted 323 household surveys with caregivers of children aged 6 to 24 months. Analysis of variance was used to estimate difference between doers and non-doers of three targeted behaviors: washing utensils with soap, keeping utensils on a raised place, and handwashing with soap. Analysis of variance analyses revealed that literacy level, ownership of animals, and presence of handwashing facility and dish racks were contextual factors predicting storage of utensils on an elevated place and handwashing frequencies. Psychosocial factors, such as time spent to wash utensils with soap, distance to the handwashing facility, and cost for soap, had an influence on washing utensils and handwashing practices. Perceived vulnerability determined effective handwashing and storage of utensils. Perceived social norms and ability estimates were favorable for the three targeted behaviors. Promotion of already existing targeted beneficial behaviors should be encouraged among caregivers. Risk perceptions on storage of utensils and handwashing practices should be increased with motivational exercises such as paint games. Caregivers’ technical know-how of local dish rack and tippy tap construction is essential.

## INTRODUCTION

Worldwide, the lives of approximately 525,000 children are lost each year from 1.7 billion cases of childhood diarrhea with the highest mortality rates reported among children aged less than 2 years in south Asia and sub-Saharan Africa.^1,2^ Furthermore, it has been reported that 550 million people fall ill, whereas 230,000 die every year globally because of diarrheal diseases associated with food contamination.^3^ Epidemiological data indicate that food could be more important than water in transmitting diarrheal disease,^4–6^ and it is estimated that 40% of the burden of foodborne disease lies with children aged less than 5 years in low- and middle-income countries. This corresponds with reports that at least 70% of diarrhea-related pathogens among children could be caused by contaminated food.^7,8^

If children aged between 0 and 6 months are exclusively breastfed, they are expected to be free from pathogens.^7^ Nevertheless, such protection is temporary because children are subsequently exposed to pathogens when introduced to complementary food between the ages of 4 and 6 months.^9,10^ This exposure together with increased environmental interaction have been linked to the high incidence of diarrhea among children aged between 6 and 24 months.^2,11–13^ To reduce diarrhea among children, the WHO has indicated important parameters that need to be implemented at the household level, including access to safe water, improved sanitation facilities, exclusive breast feeding, hygienic weaning practices, and improved personal and household hygiene.^14^

Food can become microbiologically contaminated if prepared under unhygienic conditions, and studies have shown that utensils, such as spoons, cups, pots, baby bottles, and plates, are potential sources of pathogens (such as *Escherichia coli*, *Salmonella*, and *Vibrio cholerae*) in food.^15,16^ Contamination of utensils was attributed to the method of cleaning, resulting from repeated use of wash water and dirty cloths. Because of the risk of post-cooking contamination, the cleaning of utensils before eating, particularly for high-risk groups, is integral to food safety, as demonstrated by studies in Thailand and Mali.^17,18^ As such, effective cooking of food cannot be considered as a sole critical control point, but must be combined with washing of utensils with soap and handwashing with soap at critical times.^18^

A study conducted in Bangladesh showed that caregivers have adequate knowledge of the importance of storing food and utensils on an elevated surface.^19^ However, very few translate the hygiene knowledge into practice.^20^ Imparting knowledge alone about food contamination pathways to caregivers has been found to be redundant and does not lead to associated changes in behavior. However, improving caregivers’ perceptions while building awareness about food hygiene practices has been recommended as one of the most effective approaches to achieve positive and sustained change.^21^ Contamination can also be compounded by people living in close proximity with animals. This increases the risk of food contamination if there is poor storage of utensils and leftover food, and the situation is worsened with poor handwashing practices following contact with animal and animal feces.^22,23^ Previous studies conducted in Malawi showed that food is contaminated by utensils and hands during post-cooking activities.^24,25^

Recent studies have indicated the importance of handwashing in diarrheal disease reduction, with systematic reviews showing that handwashing with soap alone can reduce diarrhea incidence by 30–47%.^26,27^ In Brazil and Bangladesh, studies have shown that poor hand hygiene practices during food preparation were a source of food contamination.^28,29^ Because handwashing has proven to effectively contribute to enteric pathogen reduction, it is important to understand the psychosocial factors that drive handwashing practices and the context in which they occur.

Changing an individual’s behavior is a process that requires change in contextual and psychosocial factors that predict human behavior in a given setting, such as attitudes, norms, and self-regulation attributes.^30^ Based on our literature review, no detailed assessment of contextual and psychosocial factors for food hygiene practices has been conducted to identify critical factors to be addressed by a behavior change intervention for the targeted area of this formative study. Psychosocial factors have been defined as the influence of social factors on an individual’s mind or behavior, and the interrelation of behavioral and social factors.^31^ Contextual factors refer to the environment in which the behavior occurs, and they include the personal (e.g., age and literacy), social (e.g., economic conditions), and physical parameters (e.g., presence of sanitation facilities such as handwashing facility).^32^

The Risk, Attitude, Norms, Ability, and Self-regulation (RANAS) model^33^ which was developed based on psychological theories^34,35^ and has been applied in this formative study was designed to understand contextual and psychosocial parameters of individuals associated with their water, sanitation, and hygiene (WASH) practices. The model presents five “factor blocks” that should be applied to understand psychosocial factors of a study population to determine a specific behavior.

### Risk factors.

The risk factors respond to the level of understanding and awareness of the person’s vulnerability and severity of diseases. They also include health knowledge about disease transmission, prevention options, and personal consequences.

### Attitude factors.

Attitude factors include beliefs about the costs and benefits of a particular behavior and feelings associated with the behavior.

### Normative factors.

The norm factors address the perception of what behavior is performed in the society and the level of personal obligation to a specific behavior. It includes how family and community members, including leaders, approve or disapprove a particular behavior.

### Ability factors.

Ability factors assess an individual’s capacity to perform a certain behavior, which includes its uptake, maintenance, and recovery from drawbacks.

### Self-regulation.

The self-regulation factors check on an individual’s plan on how to sustain a specific behavior, and they include the element on how to deal with barriers to the implementation of the behavior.

To our knowledge, the RANAS model has not been previously applied in a food hygiene assessment. However, it has been successfully used to evaluate water treatment, sanitation, and handwashing behavior.^36,37^ The RANAS model is applied in two stages: 1) determine the behavior factors for the individuals under study and 2) select behavior change techniques (BCTs) that should be applied to the identified gaps.^38^ Consequently, this can provide scientific guidance on which strategies to follow during an intervention. Because human behavior occurs in an environmental setting where a number of factors come into play, understanding of psychosocial factors alone may not be enough to bring about behavior change. As such, this must be complemented with details of the contextual factors in which the behavior occurs.

The objective of this formative study was to describe the situation and behavior, and to determine the contextual and psychosocial factors associated with 1) washing of household utensils with soap, 2) storing of household utensils on an elevated area, and 3) washing hands with soap at critical times. This study was a component of a larger body of work to understand behavioral factors related to complementary food hygiene in the development of an intervention trial.

## MATERIALS AND METHODS

### Study area.

The formative study was conducted in three rural administrative Traditional Authorities of Masache, Ngowe/Ngabu and Maseya in Chikwawa district in southern Malawi. During the 2018 population census, the Chikwawa district population was 564,684,^39^ and Chichewa is the main language of the area. Chikwawa is in a low-lying area and, therefore, prone to flooding in the rainy season. Similar to other districts of Malawi, Chikwawa has two seasons per year, that is, rainy/farming season that lasts from November to April and dry/off farming season from May to October. The district has an annual average temperature of 25.7°C and an average rainfall of 797 mm.^40^

Three Traditional Authorities were chosen taking into account their geographic location (rural remote area), socioeconomic variability (low-income communities), and access to protected water sources and high sanitation coverage (declared open defecation free), but with a continued high risk of cholera and diarrhea. Households in the targeted villages are very close to each other, and this provides an opportunity for communities to have common values and share WASH, including food hygiene issues without social resistance.

### Study population and sampling.

This formative study targeted caregivers and their children aged 6 to 24 months. In this article, the word caregiver includes any household member, including parents who are responsible for daily care of the targeted child. This includes feeding and preparing the child’s food, bathing, and assisting the child during defecation. With the use of information from the community health workers’ (i.e., locally known as health surveillance assistants) register, a list was drawn up of households with children aged between 6 to 24 months, having a pit latrine, and with access to safe water within a distance of 500 m. A sample size of 295 was calculated based on Chikwawa district diarrhea prevalence of 26.3% with an acceptable error margin of 5%.^41^ Taking into account of nonresponse rate and missing data, the sample size was increased to 323.

### Data collection.

The research team collected data from February to July 2017. As behaviors are determined by a wide range of factors, it was necessary to use different data collection methods to reveal the complexity of the socioeconomic, cultural, and other factors that influence the child caregiver’s decision on the “what,” “how,” “when,” and “why” of infant and child feeding practices. Therefore, formative data were collected from four complementary phases which included checklist and structured observations, in-depth interviews, household surveys (i.e., demographic and RANAS questions), and focus group discussions. This article presents only findings from household survey. All households (323) undertook the combined demographic and RANAS model–based household questionnaire. Initially, before conducting household surveys, observations were conducted which identified three critical behaviors: 1) washing utensils with soap, 2) keeping utensils on an elevated area, and 3) handwashing with soap at critical times, where critical times in this article mean handwashing with soap before food preparation; before eating, including child feeding; after changing child’s nappy; and after latrine use by the caregiver.

The identified three critical behaviors noted during observations were further assessed for the contextual and psychosocial factors using the RANAS model–based household questionnaire (*n* = 323) which was translated into the local language of Chikwawa district (Chichewa). Responses to the RANAS questions were recorded on a 5-point Likert scale (ranging from “not at all” to “very much” scale). The household survey questionnaire was mainly composed of closed questions that captured information about demographics, child feeding, health status and awareness, psychosocial factors related to washing utensils with soap, keeping utensils on elevated area, and handwashing with soap at critical times (example item in Table 1). Furthermore, the questionnaire contained rapid spot checks related to sanitation and hygiene structures which could be objectively observed.

**Table 1:** Risk, Attitude, Norms, Ability, and Self-regulation model–based questionnaire (e.g., factors and items for washing utensils with soap)

Behavior determinants	Selected items
Risk factors	
Vulnerability	In general, how high do you think is the risk that you get diarrhea?
Severity	Imagine that you contracted diarrhea. How severe would be the impact on your life in general?
Health knowledge	Can you tell me what causes diarrhea? Could you please tell me if each of the following is a cause or not? For example, no handwashing with soap after defecation. Could you please tell me for each whether it is a preventive measure for diarrhea or not? For example, drink treated water
Attitudinal factors	
Belief—effort	How pleasant is it for you to wash kitchen utensils with soap and water?
Belief—time-consuming	How time-consuming is it to wash kitchen utensils with soap and water?
Belief—expensive	How expensive is it for you to always wash kitchen utensils with soap and water?
Feelings	How much do you like always washing kitchen utensils with soap and water?
Normative factors	
Others’ behavior household	How many people of your household always wash kitchen utensils with soap and water?
Others’ behavior village	How many people of your village always wash kitchen utensils with soap and water?
Others’ approval	People who are important to you like your family members, friends, Non Governmental Organization (NGO) workers, or pastor, how much do they approve that you always wash kitchen utensils with soap and water?
Personal obligation	How strong do you feel a personal obligation to yourself to always wash kitchen utensils with soap and water?
Ability factors	
Confidence in performance	How confident are you that you can always wash kitchen utensils with soap and water?
Difficult water	How difficult is it to always get water for washing kitchen utensils?
Barriers hurry	Imagine that you are in a hurry, for example, because you want to go for relief distribution: How confident are you that you can always wash kitchen utensils with soap and water?
Self-regulation factors	
Coping plan	Do you have a plan what to do so that you always have soap for washing kitchen utensils? Plan, please specify.
Remembering (pay attention)	How much do you pay attention to washing utensils with soap and water?
Remembering (forgetting last 24 hours)	When you think about the last 24 hours: How often did it happen that you forgot to wash kitchen utensils with soap and water?
Commitment (important)	How important is it for you to wash kitchen utensils with soap and water?
Washing utensils with soap behavior	How often do you wash kitchen utensils with soap?

Response scales: 5-point Likert scale (from “not at all” to “very much”; from “at no time” to “almost each time”; from “never” to “very often”; and from “nobody” to “almost all of them”).

Household survey data collection was conducted by 10 well-trained and experienced research assistants who were fluent in the local language (Chichewa). Pretesting of the questionnaire was conducted before data collection where the research team identified and eliminated irrelevant questions, whereas key questions were further edited for easy understanding.

### Data analysis.

Demographic household and RANAS data were collected using Open Data Kit software (Department of Computer Science and Engineering, University of Washington, Seattle, WA) on android tablets and exported to Microsoft Excel (Microsoft corporation, Redmond, WA) and quality checked before being exported to Statistical Package for Social Sciences (SPSS) where frequency distribution of demographic characteristics using descriptive statistics was plotted. IBM SPSS version 25, the PROCESS macro for SPSS, was used to undertake all statistical tests (IBM, Armonk, NY). The household RANAS model–based data were analyzed using ANOVA mean comparison analysis to determine the differences between doer and non-doer contextual and psychosocial factors for the targeted behaviors. To measure the three targeted behaviors, data collectors asked caregivers how often they washed utensils with soap, how often they kept utensils on a raised place, and how often they washed hands with soap at critical times. Frequencies were measured on a 5-point scale. All factors falling at or below the mid 3-point value on a scale of 1–5 were considered non-doers of the targeted behaviors, whereas those factors at or above 4 were doers of the behavior, and the mean score for each targeted behavior was calculated. Washing utensils with soap, keeping utensils on an elevated area, and handwashing with soap were dependent variables, whereas behavioral factors of the RANAS model were independent variables. Three questions were asked to caregivers to assess knowledge about diarrheal disease causation, signs, and preventive measures. The ratio of correct answers from the caregivers to all possible answers formed the health knowledge constructs. A single item was used to measure perceived severity, whereas perceived vulnerability of diarrhea and other psychosocial factors were measured with multiple items. The WHO and United Nations Children’s Fund definition of diarrhea was used when assessing diarrhea incidence among targeted children.^11^ For each targeted behavior, the significant factors among those noted with ANOVA calculation were further analyzed (i.e., any factor at *P* < 0.05 using ANOVA) with effect size, *d*, where Cohen’s *d* values mean small for those = or < 0.20, medium = or < 0.50, and large = or > 0.80.

### Ethics.

The formative study protocol was approved by the University of Malawi’s College of Medicine Research Ethics Committee (P.04/16/1935). Permission was obtained from the local authorities, that is, Chikwawa district council, Chikwawa district health office, and the traditional chiefs. The participants were informed of the research objectives and were advised that they had the freedom to refuse participation or withdraw from the study at any time. Participants’ written informed consent was obtained before inclusion in the study. Participants were provided with a unique identifying number, and data were anonymized during data analysis. Data were accessed only by the authors. The study was registered with the Pan African Clinical Trials Registry (PACTR201703002084166).

## RESULTS

### Sociodemographic characteristics.

All respondents of the household questionnaire were females whose age ranged from 18 to 53 years (mean 26.72 with SD 6.78). The majority of them (71%) attended primary education, whereas 16% had never been to school. Income was primarily from subsistence farming (67%), and majority of the households (74%) earned at most $14 per month. As such, households reported some levels of uncertainty about food supply. The age range of targeted children was 6–24 months (mean 14.27 with SD 5.72) of which 49% were females. Forty percent of children were introduced to complementary food (i.e., porridge from maize flour) when they were between 3 and 6 months old, and 27% of the targeted children were reported to have had diarrhea in the 2 weeks before the survey. No participating households were connected to an electrical power supply, and therefore, none owned a refrigerator. Domesticated animals, such as pigs, dogs, goat and poultry, were observed roaming freely in household yards. Human and animal feces were observed in 2% and 52.9% of the household yards, respectively.

Caregivers accessed safe water through boreholes (93%) and piped water supply (3%). Latrines were owned by 95% of the households, whereas 5% either depended on their neighbors’ latrine or practiced open defecation. Despite high coverage, most toilets were unimproved traditional latrines (64%) subject to collapse during the rainy season and offering minimal privacy. Soap was available in 61% of the households, and it was prioritized in the following order: washing clothes, bathing, washing kitchen utensils, and handwashing.

### Contextual factors: doer versus non-doer analysis.

Contextual factors were compared between doers and non-doers of the three targeted behaviors. Statistical analysis identified significant variables related to handwashing with soap and keeping of utensils on an elevated place, whereas no significant variables were observed for washing utensils with soap (Table 2). Factors that were found to be significant for handwashing included level of literacy, where those who were literate washed hands with soap at critical times more frequently than those who were not literate (doers = 50%; non-doers = 38%). Similarly, caregivers who had handwashing facilities reported to wash hands with soap more than those who had no handwashing facilities (doers = 59%; non-doers = 46%). On keeping utensils, caregivers who had domestic animals kept their utensils more frequently on an elevated place than those who had no animals (doers = 78%; non-doers = 60%), and those who had locally made dish racks kept their utensils more on an elevated place than those who had no dish racks (doers = 75%; non-doers = 14%).

**Table 2:** Comparison of contextual factors of the study participants on washing of utensils with soap, keeping utensils on a raised place, and handwashing with soap

Variable	Scale	Washing utensils with soap		Keeping utensils on raised a place		Handwashing with soap	
		Doer	Non-doer	Doer	Non-doer	Doer	Non-doer
Literacy	Yes/No	47%	40%	47%	42%	50%*	38%*
Marital status	Yes/No	86%	87%	84%	87%	85%	87%
Age in years *mean* (SD)	–	25.73 (6.0)	27.60 (7.3)	27.18 (6.9)	26.55 (6.8)	25.46 (6.2)	27.57 (7.0)
Owned land for farming	Yes/No	82%	84%	78%	85%	83%	84%
Owned livestock	Yes/No	69%	62%	78%*	60%*	68%	63%
Presence of bicycle	Yes/No	64%	61%	69%	60%	66%	60%
Presence of radio	Yes/No	40%	40%	44%	38%	39%	40%
Presence of handwashing facility	Yes/No	–	–	–	–	59%*	46%*
Presence of dish rack	Yes/No	–	–	75%†	14%†	–	–

* *P* ≤ 0.05.

† *P* ≤ 0.001; *N* = 323.

### Psychosocial factors: washing of household utensils.

From the household spot checks, the study noted that 29% of the caregivers washed their utensils with soap. Risk, Attitude, Norms, Abilities, and Self-regulation model–based questions were asked to understand psychosocial factors that contributed to caregivers not using soap when washing utensils. As shown in Table 3, we did not find significant differences between doers and non-doers on vulnerability, severity, health knowledge, attitude (effort), personal obligation, and commitment (importance). As such, these factors should not be the focus for a behavior intervention. Significant differences with medium to high cohen’s *d* values were found on others’ behavior (relatives; *d* = 0.64), others’ approval (*d* = 0.74), and confidence in performance (continuation—barrier water; *d* = 0.7), where non-doers reported highly that they could not wash utensils with soap because of inadequate water at the household (Table 3). This means that these factors should be key targets for behavior change among non-doers of washing utensils with soap. Medium effect was found in the attitude factor “pleasant” (*d* = 0.45) and self-regulation (remembering; *d* = 0.44), meaning that doers found it more pleasant to wash utensils with soap than non-doers. Similarly, the doers were less likely to forget to wash their utensils with soap than the non-doers. Slightly significant differences between doers and non-doers were noted on others’ behavior (village; *d* = 0.36), intention (*d* = 0.33), and communication (*d* = 0.3). This implies that non-doers do not desire much to wash their utensils with soap than the doers. In addition, the non-doers do not discuss much with their friends or relatives about the practice of washing utensils with soap compared with the doers.

**Table 3:** Washing of utensils with soap: doer and non-doer Risk, Attitude, Norms, Ability, and Self-regulation psychosocial factors’ mean compared with analysis of variance

Factors group	Behavioral factors	Doers, *M* (SD)	Non-doers, *M* (SD)	Cohen’s *d*
Risk factors	Vulnerability	4.06 (1.39)	3.80 (1.48)	n.s.
	Severity	4.36 (0.97)	4.24 (1.06)	n.s.
	Health knowledge	9.29 (3.09)	8.83 (3.22)	n.s.
Attitude factors	Pleasant†	4.8 (0.65)	4.38 (1.15)	0.45
	Time‡	1.14 (0.41)	1.3 (0.82)	0.25
	Effort	1.13 (0.64)	1.22 (0.66)	n.s.
Norms	Others’ behavior relatives†	3.26 (1.3)	2.51 (1.02)	0.64
	Others’ behavior village‡	2.88 (1.01)	2.54 (0.9)	0.36
	Others’ approval†	4.79 (0.51)	4.2 (1.01)	0.74
	Personal obligation	2.42 (1.86)	2.37 (1.73)	n.s.
Ability factors	Ability (confidence in performance [continuation]—barrier: water)†	4.33 (0.97)	3.57 (1.2)	0.7
Self-regulation factors	Commitment (importance)	4.88 (0.57)	4.79 (0.67)	n.s.
	Remembering (forgetting)†	2.61 (1.16)	3.12 (1.15)	0.44
Additional factors	Intention‡	3.82 (1.4)	3.34 (1.52)	0.33
	Communication‡	3.14 (1.42)	2.73 (1.32)	0.3

n.s. = not significant.

*N* = 323; washing of utensils with soap: doers *N* = 154 and non-doers *N* = 169. All questions (excluding knowledge questions, which were sum score) included a 5-point Likert scale and response choices from “1—not at all” to “5—very much.”

† *P* ≤ 0.001.

‡ *P* ≤ 0.01.

### Psychosocial factors: storage of clean utensils.

The study found that 31% of the caregivers kept their utensils on an elevated place that could not easily be reached by animals. On psychosocial factors related to storage of utensils on an elevated place, significant differences between doers and non-doers could not be found on severity, health knowledge, attitude (time and effort), and personal obligation (Table 4). Hence, these factors should not be prioritized for intervention. However, statistical differences on cohen’s *d* values were noted on others’ behavior (relatives; *d* = 0.71 and village; *d* = 0.82), others’ approval (*d* = 0.6), and confidence in performance which included “difficult” (*d* = 0.44), “hurry” (*d* = 57), and “restart” (*d* = 0.65) (Table 4). This implies that non-doers perceived that people in their village, including their relatives, do not keep their utensils on an elevated place. In addition, the non-doers were unlikely to restart or continue keeping utensils on a raised place if they stopped for other reasons and found it more difficult to keep or dry their utensils on a raised place if they do not have a dish rack. The non-doers also perceived that they communicate less with others (*d* = 0.47) about using an elevated surface to keep or dry their utensils and felt less vulnerable (*d* = 0.58) to the risk of diarrheal disease than doers, which is related to the non-doers perception that keeping utensils on a raised place is not a pleasant practice (*d* = 0.35).

**Table 4:** Keeping of utensils on an elevated position: doer and non-doer Risk, Attitude, Norms, abilities, and Self-regulation psychosocial factors’ mean compared with analysis of variance

Factors	Behavioral factors	Doers, *M* (SD)	Non-doers, *M* (SD)	Cohen’s *d*
Risk factors	Vulnerability†	4.19 (1.27)	3.35 (1.61)	0.58
	Severity	4.37 (0.94)	4.27 (1.04)	n.s.
	Health knowledge	8.78 (2.85)	9.15 (3.27)	n.s.
Attitude factors	Pleasant‡	4.69 (0.88)	4.33 (1.23)	0.35
	Time	1.28 (0.82)	1.21 (0.7)	n.s.
	Effort	1.08 (0.49)	1.21 (0.7)	n.s.
Norm factors	Others’ behavior relatives†	2.99 (1.27)	2.16 (1.06)	0.71
	Others’ behavior village†	2.92 (0.99)	2.19 (0.79)	0.82
	Others’ approval†	4.49 (0.8)	3.83 (1.22)	0.6
	Personal obligation	2.58 (1.92)	2.4 (1.73)	n.s.
Ability factors	Confidence in performance (difficult)†	4.25 (1.34)	3.61 (1.56)	0.44
	Confidence in performance (hurry)†	4.16 (1.42)	3.26 (1.74)	0.57
	Confidence in performance (restart)†	4.63 (0.9)	3.81 (1.54)	0.65
Self-regulation	Commitment (importance)	2.60 (1.94)	2.20 (1.73)	n.s.
	Remembering (forgetting)	1.06 (0.47)	1.19 (0.5)	n.s.
Additional factors	Communication†	3.19 (1.4)	2.53 (1.41)	0.47

n.s. = not significant*.*

*N* = 323; Keeping of utensils on a raised place: doers *N* = 88 and non-doers *N* = 235. All questions (excluding knowledge questions, which were sum score) included a 5-point Likert scale and response choices from “1—not at all” to “5—very much.”

† *P* ≤ 0.001.

‡ *P* ≤ 0.01.

### Psychosocial factors: handwashing with soap at critical times.

A specific place for handwashing was found in 51% of the households, of which 62% were located near the latrine. However, only 19% of the handwashing facilities had soap and water. The study explored psychosocial factors that contributed to nonuse of soap when washing hands at critical times. As shown in Table 5, the highest associated population effect sizes for handwashing with soap at critical times were attitude (like; *d* = 1.17) and confidence in continuation (*d* = 0.81). This implies that non-doers show a lower preference to washing their hands with soap and are less likely to continue using soap when washing hands at critical times. Furthermore, the non-doers found it expensive (soap; *d* = 0.56) and time-consuming (time; *d* = 0.45) to wash hands with soap compared with the doers. The factor attitude (distance) was also found to be significant (*d* = 0.34). As such non-doers perceived that the handwashing facility located near the latrine was too far for them to wash hands with soap during other critical times of handwashing, such as before preparing food. The caregivers found it hard to have another handwashing facility within the cooking area because they had no technical know-how on handwashing facility construction. They depended on their husbands to construct the handwashing facilities, but they, in most cases, were reportedly engaged with food-fetching activities for the home. Other significant factors included cost (*d* = 0.27), others’ behavior (relatives; *d* = 0.76 and village *d* = 0.54), remembering (*d* = 0.7), and communication (*d* = 0.62). Furthermore, risk factors (vulnerability and severity) were slightly significant for handwashing with soap practice (*d* = 0.26 and 0.28, respectively). This means that doers found it more probable that they would suffer from diarrhea and its severity would be more, compared with the non-doers, although health knowledge, time, effort, confidence in performance (water), and commitment (importance) were insignificant.

**Table 5:** Handwashing at critical times: doer and non-doer Risk, Attitude, Norms, Abilities, and Self-regulation psychosocial factors’ mean compared with analysis of variance

Factors	Behavioral factors	Doers, *M* (SD)	Non-doers, *M* (SD)	Cohen’s *d*
Risk factors	Vulnerability*	4.36 (1)	4.08 (1.16)	0.26
	Severity*	4.46 (0.86)	4.18 (1.1)	0.28
	Health knowledge	9.4 (3.25)	8.8 (3.09)	n.s.
Attitude factors	Time	1.07 (0.47)	1.16 (0.62)	n.s.
	Effort	1.11 (0.57)	1.17 (0.72)	n.s.
	Distance†	1.41 (1.2)	1.9 (1.61)	0.34
	Cost*	1.55 (1.23)	1.92 (1.46)	0.27
	Handwashing removes germs‡	4.86 (0.51)	4.46 (1)	0.5
	Like‡	4.43 (1.03)	3.05 (1.31)	1.17
Norm factors	Others’ behavior relatives‡	3.9 (1.24)	2.89 (1.47)	0.76
	Others’ behavior village‡	2.9 (1)	2.37 (0.96)	0.54
	others’ approval‡	4.76 (0.68)	4.43 (1.03)	0.38
Ability factors	confidence in performance (sure)‡	4.69 (0.79)	3.7 (1.54)	0.81
	confidence in performance (water)	1.11 (0.62)	1.11 (0.54)	n.s.
	confidence in performance (soap)‡	1.57 (1.19)	2.35 (1.59)	0.56
	confidence in performance (time)‡	1.05 (0.29)	1.38 (1)	0.45
Self-regulation	remembering (forgetting)‡	1.73 (1)	2.66 (1.6)	0.7
	commitment (importance)	4.86 (0.49)	4.85 (0.55)	n.s.
Additional factors	communication‡	3.39 (1.39)	2.55 (1.31)	0.62

n.s. = not significant.

*N* = 323; handwashing with soap at critical times: doers *N* = 132 and non-doers *N* = 191. All questions (excluding knowledge questions, which were sum score) included a 5-point Likert scale and response choices from “1—not at all” to “5—very much.”

* *P* ≤ 0.05.

† *P* ≤ 0.01.

‡ *P* ≤ 0.001.

### Selection of the behavior change techniques.

Based on the results from formative data, the RANAS model fact sheet^33^ provided guidance on which BCTs should be applied for the behavioral interventions. Evidence-based decisions in the choice of BCTs to promote complementary food hygiene practices were derived from analysis of contextual and psychosocial factors. Furthermore, household spot checks noted that only 29% and 31% of the visited households had soap for washing utensils and had an elevated place for keeping kitchen utensils, respectively, whereas handwashing facilities with soap and water were noted in 19% of the households. The formative data provided a platform for developing interventions with an overall aim of promoting child caregivers toward improved complementary food hygiene practices. As shown in Table 6, the strategies considered for the interventions would aim the following. 1) Build awareness on complementary food hygiene habits at an individual and community level. 2) Reinforce the ability to wash hands with soap at all critical times, wash utensils with soap, and keep them on an elevated place. Thus, interventions to improve infrastructure (i.e., dish racks and handwashing facilities) are being suggested to boost caregiver’s self-efficacy and, therefore, increase their confidence to perform the behaviors.^42^ In addition, their confidence in performance would be enhanced through demonstrations such as “Glo germ gel” and “hand and utensil painting exercise” (see the following paragraphs) that would lead to an increased perception of self-efficacy. 3) Indicate that others are already performing the desired practices. Thus, public commitment to show that others are performing the targeted behaviors would be performed through open days where caregivers would also sing songs about targeted behaviors. Public pledges would enhance normative factors and posters to be placed outside caregivers’ houses would show community members that others are performing targeted behaviors that would boost descriptive norms. 4) Reinforce the action self-efficacy through use of attractive posters with key messages to remind caregivers to always wash utensils and hands with soap will enhance their confidence to practice the behavior.

**Table 6:** Translation into practical strategies

RANAS factor blocks	Behavior	Target RANAS behavioral determinants	Definitions of the behavioral determinants	Intervention types	Corresponding RANAS behavior change technique	Practical strategies
Risk factors	Keeping utensils on elevated area and handwashing with soap	Perceived vulnerability	Perception of the seriousness of suffering from diarrhea	Information interventions	Provide practical information on behavior and health outcomes	Create practical information exercises and posters
Norm factors	Washing utensils with soap, keeping utensils on raised place, and handwashing with soap	Descriptive and injunctive norms	Perception of other caregivers performing the three behaviors	Normative interventions	Flag out norms	Public commitment event through open days and cluster meetings. Role-model guided practice. Create posters
Ability factors	Washing utensils with soap	Action self-efficacy	Certain to always wash utensils with soap	Ability interventions	Increase confidence in behavior: prompt guided practice	Create practical exercises
	Keeping utensils on raised place	Action self-efficacy	Certain to always be able to keep utensils on a raised place	Infrastructure and ability interventions	Increase confidence in behavior: performance by providing practical instructions	Provide practical instructions on dish rack construction and show pleasantness of the behavior
	Handwashing with soap	Action self-efficacy	Certain to always be able to wash hands with soap at four critical times	Infrastructure and ability interventions	Increase confidence in behavior: performance by providing practical instructions	Create games and provide practical instructions on tippy tap construction
Self-regulation factors	Handwashing with soap	Action self-efficacy	Certain to always be able to wash hands with soap	Remembering intervention	Memory aids and environmental prompts	Create memory aids for handwashing

RANAS = Risk, Attitude, Norms, Ability, and Self-regulation.

## DISCUSSION

As reported in other developing countries, complementary food hygiene is suboptimal in Malawi,^18,21,43–45^ and high prevalence of diarrhea among children in this study suggests that food hygiene practices such as these may play an important role in child health. However, motivators and barriers for food hygiene improvements in this setting were not clearly understood. For the first time, our study assessed the contextual and psychosocial factors related to caregivers’ food hygiene practices in rural Malawi. Such data were necessary for the development of population-tailored behavior change interventions. In this formative study, data were collected from child caregivers who had children aged between 6 and 24 months in Chikwawa, Malawi. Normative factors about others’ behavior and ability factors were identified as the main factors for all three behaviors. In addition, the self-regulation factor (remembering) was found to be a strong predictor of handwashing with soap at critical times. Guided practice, memory aids, information about others’ behavior, and model behavior are being considered in a behavior change intervention for improved practices on washing of utensils with soap, keeping utensils on a raised place and handwashing with soap at critical times.

Although we acknowledged that washing utensils without soap is not the only risk factor for diarrhea among children, the practice of washing utensils without soap could increase the risk of food contamination as this is a proven route of pathogen transmission.^15,16^ As such, the creation of effective promotion strategies to encourage the use of soap to wash utensils is important. The practice of placing utensils on the ground before, during, and after washing utensils is common in Malawi and may increase the risk of childhood diarrhea contaminating utensils with pathogens in soil and animals (directly and via feces). Nevertheless, this study found that the presence of a dish rack at a household influenced the doers to keep their utensils on an elevated place compared with the non-doers. Previous research has shown that promoting existing beneficial behavior is important in addressing local needs.^19^ Thus, the safe practice of using locally made dish racks which is already performed by a few (31%) in the study area should be promoted.

In this study, use of soap for handwashing was uncommon. Soap was found to be prioritized for other household usage such as washing clothes and bathing. Contrary to what was reported by Seimetz et al.,^46^ purchase of soap in this study was found to slightly influence handwashing with soap practice. However, usage of soap greatly depended on influence from others and the availability of a convenient place for handwashing. Failure of caregivers to wash hands because of the lack of a handwashing facility confirms what has been previously reported that a specific place for handwashing is a predictor of household handwashing frequency.^47,48^ Generally, 62% of the handwashing facilities were located near the latrine (behind and away from the cooking area) which affected the frequency of caregivers’ washing hands at other critical times (e.g., during food preparation). Furthermore, it has been shown in this study that the presence of a handwashing facility at a household increased the handwashing practice among the doers compared with the non-doers. Thus, constructing additional handwashing facilities within the cooking area could improve the frequency of handwashing practice.

### Interpretation of results and implication for practice.

Three knowledge sections in the questionnaire showed no significant difference between doers and non-doers about diarrhea causation, signs/symptoms, and prevention as knowledge was found to be high in both groups. However, significant differences in risk perception between doers and non-doers were noticed on keeping utensils on a raised place and handwashing with soap practices. Thus, practical strategies to sensitize the caregivers to the health risks associated with storage of utensils and handwashing with soap should be incorporated in an intervention.

Washing utensils with soap, keeping utensils on an elevated surface, and handwashing with soap strongly interdepended on the normative factor—others’ behavior (i.e., relatives and friends). A study in Nepal showed that influence from others plays a major role in one’s behavior about sanitation and hygiene.^49^ Therefore, corresponding normative BCTs should be applied to facilitate behavior change. As community meetings have been reported to strengthen normative elements,^50,51^ group meetings with caregivers would be essential where a positive group identity would be reinforced and role models would be identified to promote the behaviors. In addition, communication about the behaviors among caregivers would be strengthened through the group meetings. Household visits would be conducted as follow-up to group meetings to prompt guided and behavioral practice at an individual level. Importantly, BCTs related to personal commitment would be appropriate to address personal norms toward the three behaviors. Such commitment should be made in public by caregivers together with their husbands as they have been found to have a major role in the construction of handwashing facilities and dish racks. As reported in other behavior change studies,^36,37,49^ public pledges would also help to reach out to more people and, thus, change descriptive norms. Having adequate water at the household increased the confidence of caregivers to wash utensils with soap. This suggests that promoting adequate water availability at households is a potential strategy for washing utensils with soap. Role models on this practice should be encouraged to demonstrate to others how they manage to have adequate water in their homes for washing utensils.

Caregivers’ abilities (confidence in performance) to keep utensils on an elevated place and wash utensils and hands with soap were a very strong predictor for the practice of these behaviors. The lower perceived self-efficacy in washing utensils with soap, keeping utensils on an elevated place, and handwashing with soap among the non-doers requires the implementation of a corresponding BCT. Demonstrations such as “Glo germ gel” and “hand and utensil painting exercise” could be applied to strengthen caregiver’s belief and ability to continuously use soap when washing hands and utensils as its effectiveness would be appreciated. Hand painting exercises show the potential movement of pathogens from one person to another through hand shaking and being in contact with household items, for example, utensils. Participants put paint in their palm and then shake hands among themselves and touch household items to represent spreading of germs. While having paint in the hands, some are asked to wash hands with soap, whereas others without and notice the difference. Similarly, the utensil painting exercise demonstrates the effectiveness of soap in removing dirt and germs from utensils such as plate. Handwashing Glo germ gel reveals areas in the hands that are concentrated with germs.^52^ Practical demonstrations on dish rack and hand washing facility construction should be promoted to strengthen the perception of self-efficacy, thus reinforcing ability factors. In addition, the use of behavioral cues should be incorporated to remind caregivers’ abilities to wash utensils and hands with soap. The use of such interesting and innovative approaches has proven to be effective in behavior change initiatives.^53–55^

## LIMITATIONS OF THE STUDY

Self-reported findings are prone to bias as the participants may report what the researcher wants to hear. However, this was controlled by conducting spot checks on some of the variables that were reported by the participants. Food hygiene practices cover additional practices to those covered in this article, such as storage conditions and reheating of leftover food. However, further analysis of formative research findings assessed these parameters in the same study setting. Sociocultural practices and geographical conditions across Malawi may differ; hence, the results of this study may not be applied to all the rural areas without further study. In addition, during recruitment, all study households had a latrine and access to safe water within a distance of 300 m. This is not the case with other households in rural settings of Malawi. However, despite the stated limitations, this research provides a good platform for understanding the contextual and psychosocial factors related to complementary food hygiene practices for the design of an effective food hygiene intervention in rural Malawi.

## CONCLUSION

This study for the first time has applied the RANAS model to assess contextual and psychosocial factors influencing child caregivers’ behavior relating to food hygiene practices in rural Malawi. This research provides evidence-based results as a basis for the development and implementation of food hygiene interventions to contribute toward prevention of diarrheal diseases. Selected contextual (i.e., presence of handwashing facility, locally made dish rack and ownership of animals) and psychosocial factors which include normative, ability, and self-regulation (remembering) factors have been identified as strong predictors for the success of an intervention that focuses on washing of utensils with soap, keeping of utensils on an elevated place, and hand washing with soap at critical times. Therefore, they should be considered for promotion in future initiatives.
